# Supplementary material for: ARMH3 is an ARL5 effector that promotes PI4KB-catalyzed PI4P synthesis at the trans-Golgi network
Source: Nat Commun. 2024 Nov 23;15:10168. doi: 10.1038/s41467-024-54410-y (PMC11585589; doi:10.1038/s41467-024-54410-y)
Supplement: Supplementary file 1 — Supplementary Information [file 41467_2024_54410_MOESM1_ESM.pdf]

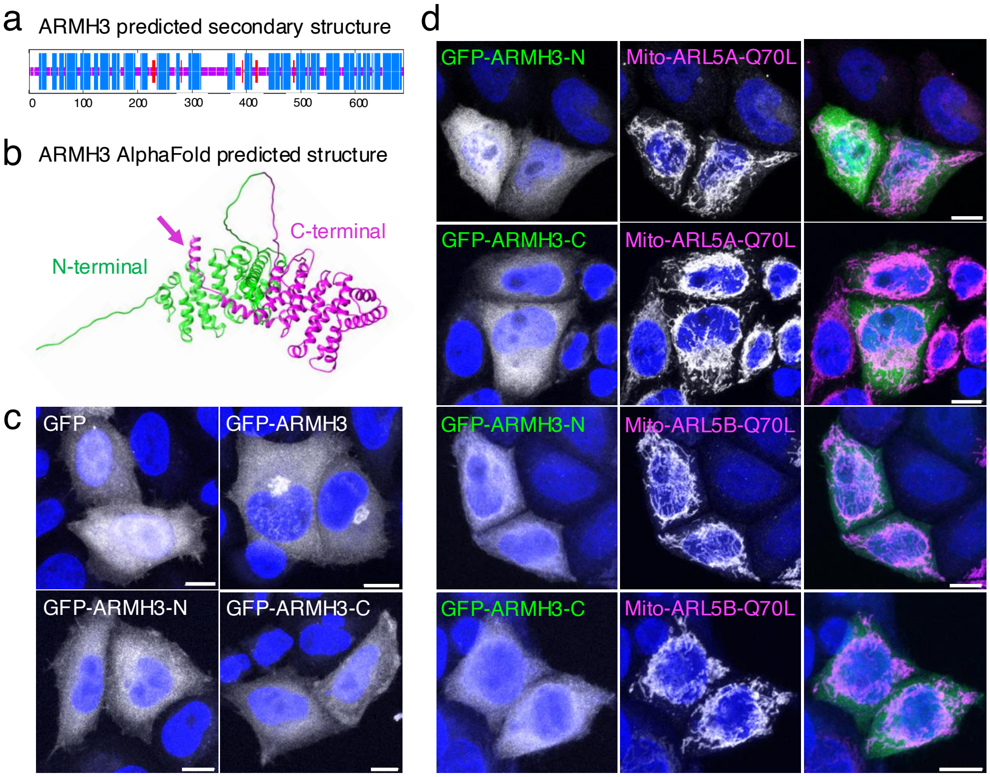

Fig. S1: Integrity of ARMH3 fold is required for Golgi localization and ARL5 binding. (a) Schematic representation of human ARMH3 consensus secondary structure predicted by the NPS@ server https://npsa.lyon.inserm.fr/cgi-bin/npsa_automat.pl?page=/NPSA/npsa_seccons.html. Regions predicted to be α-helix (blue), β-sheet (magenta), and random coil (purple) are indicated. (b) Structural model of human ARMH3 predicted by AlphaFold (https://www.uniprot.org/uniprotkb/Q5T2E6/entry). N-terminal (1-346) and C-terminal (347-689) halves are colored green and magenta, respectively, using UCSF Chimera software. (c) Fluorescence microscopy of GFP or GFP-ARMH3 (full length, N-terminal, and C-terminal halves) expressed by transient transfection in WT HeLa cells. GFP fluorescence is shown in grayscale and nuclei (DAPI) in blue. Scale bars: 10 µm. Notice the failure of the GFP-ARMH3 N- and C-terminal halves to associate with the Golgi complex. (d) Mitochondria-relocalization assays. WT HeLa cells were transiently co-transfected with constructs encoding GFP-ARMH3 (N- and C-terminal halves) (green) and the indicated Mito-ARL5 constructs (magenta). Cells were then fixed, permeabilized, immunolabeled for BioID2. Single-channel images are shown in grayscale with nuclei (DAPI) in blue, with respective double label on right. Scale bars: 10 µm. Notice the failure of N- and C-terminal halves of ARMH3 to relocalize to mitochondria.

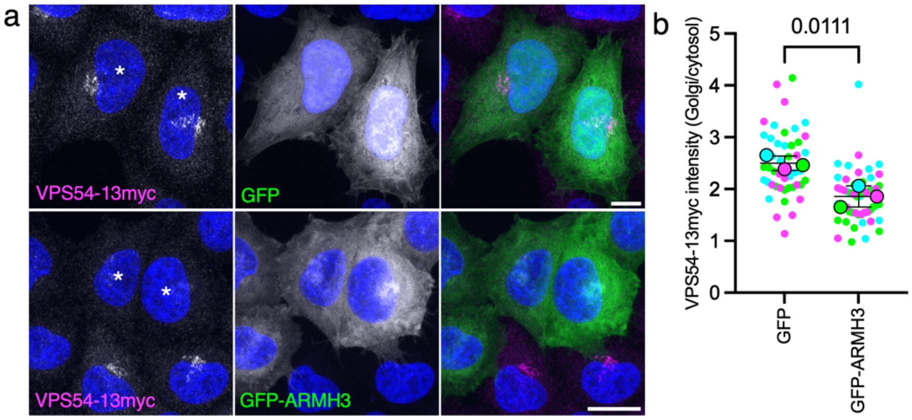

Fig. S2: ARL5 effectors ARMH3 and VPS54 compete for recruitment to the Golgi complex. (a) HeLa stably expressing VPS54-13myc transfected with plasmids encoding GFP (control) or GFP-ARMH3, fixed and stained for myc. Scale bars: 10 µm. (b) Quantification of VPS54-13myc intensity at the Golgi complex relative to the cytosol from experiments such as that shown in panel a. The graph represents the mean ± SD of values from 3 independent, color-coded experiments. The statistical significance of the differences was calculated by unpaired t-test (two-tailed). *P*-value is indicated on the graph. Notice the decrease in VPS54-13myc recruitment to the Golgi complex upon expression of GFP-ARMH3. Source data are provided in the Source Data file.

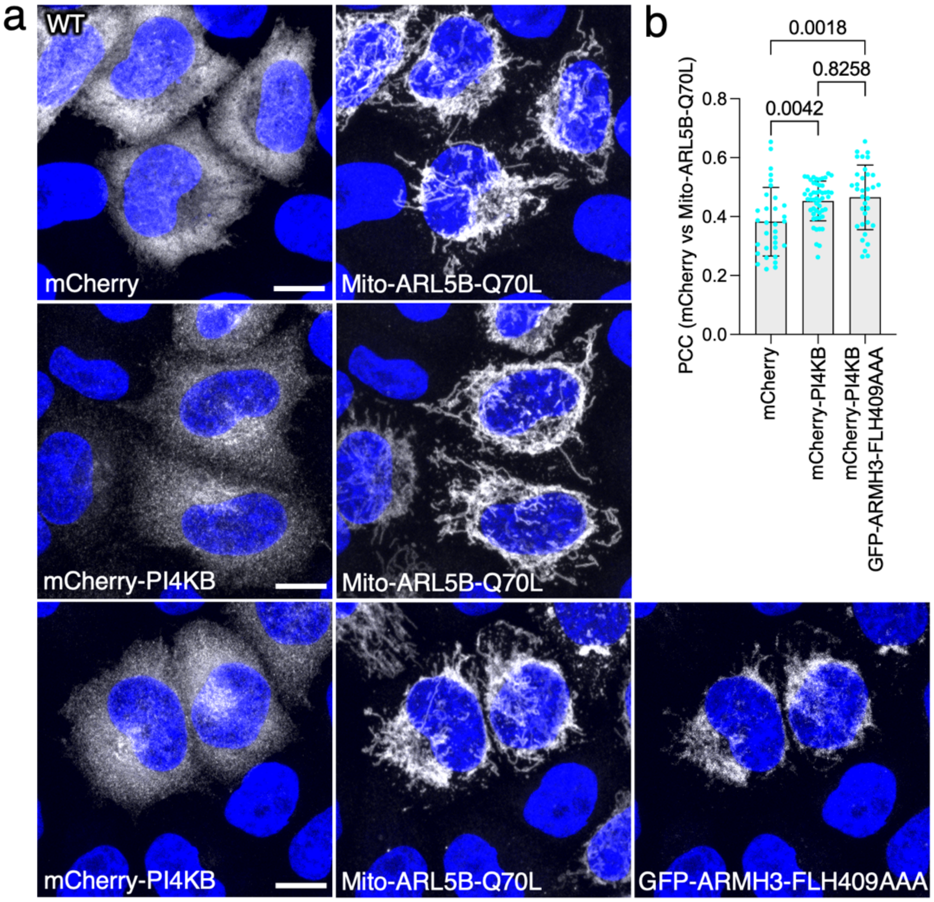

Fig. S3: PI4KB-binding mutant ARMH3 fails to enhance recruitment of PI4KB by ARL5.
(a) Mitochondria-relocalization assay. WT HeLa cells were co-transfected with plasmids encoding mCherry or mCherry-PI4K B in combination with Mito-ARL5B-Q70L, minus or plus GFP-ARMH3-FLH409AAA, as indicated in the figure. Cells were then fixed, permeabilized, immunostained for mCherry and BioID2, and imaged by confocal microscopy. Images are shown in grayscale with nuclei (DAPI) in blue. Scale bars: 10 µm. (b) Quantification of the Pearson's correlation coefficient (PCC) of mCherry *vs* Mito-ARL5B-Q70L from the experiment shown in panel a. The graph represents the mean ± SD of values from 32-51 cells. The statistical significance of the differences was calculated by one-way ANOVA with multiple comparisons using Tukey's test. *P*-values are indicated on the graph. Source data are provided in the Source Data file.

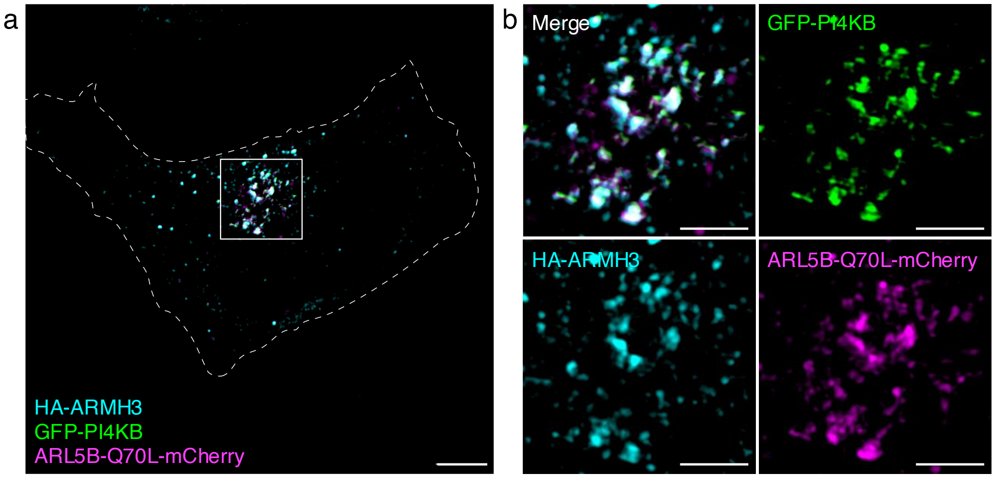

Fig. S4: Structured illumination microscopy (SIM) showing co-localization of ARL5B, ARMH3, and PI4KB at the Golgi apparatus.

(**a**) SIM of WT HeLa cell stably expressing ARL5B-Q70L-mCherry (magenta), HA-ARMH3 (cyan) and GFP-PI4KB (green). Cells were fixed, permeabilized, and immunostained for the HA epitope. Scale bar: 10 μm. (**b**) Enlarged merged and single-channel views of the boxed area from panel a. Scale bar: 5 μm. Notice the high degree of co-localization of the three proteins.

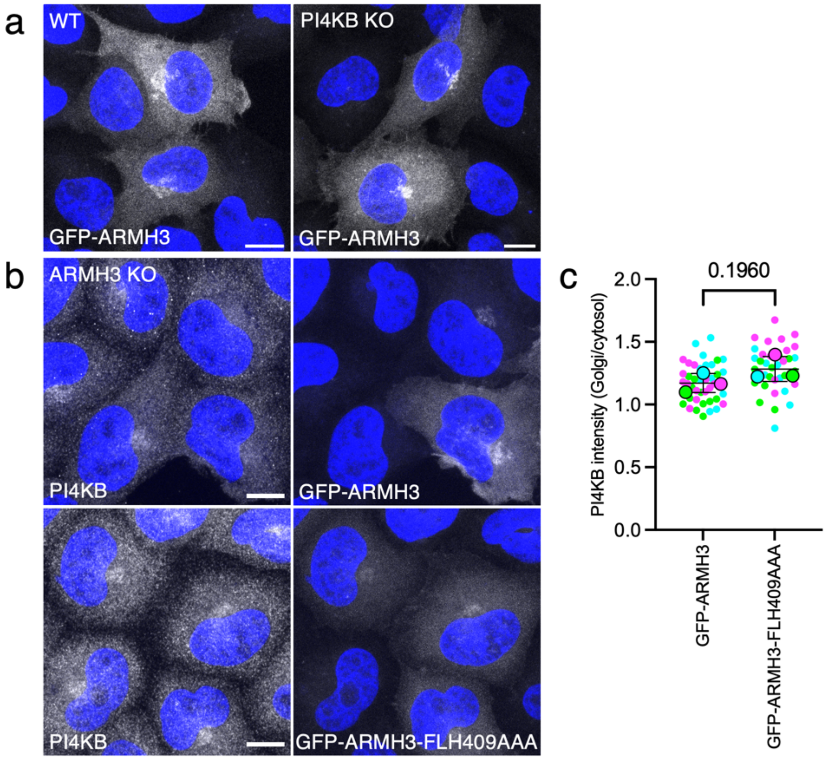

Fig. S5: ARMH3 and PI4KB are not dependent on each other for recruitment to the Golgi apparatus.

(a) Fluorescence microscopy of WT and PI4KB-KO HeLa cells expressing GFP-ARMH3. Note that GFP-ARMH3 is recruited to the Golgi apparatus in PI4KB-KO cells. (b) Immunofluorescence microscopy of ARMH3-KO HeLa cells expressing GFP-ARMH3 or GFP-ARMH3-FLH409AAA. Cells were fixed, permeabilized, and immunostained for endogenous PI4KB. Scale bar: 10 µm. (c) Quantification of PI4KB intensity at the Golgi complex relative to the cytosol from experiments such as that shown in panel b. The graph represents the mean ± SD of the means from 3 independent, color-coded experiments. The statistical significance of the differences was calculated by unpaired t-test (two-tailed). *P*-value is indicated on the graph. Source data are provided in the Source Data file.

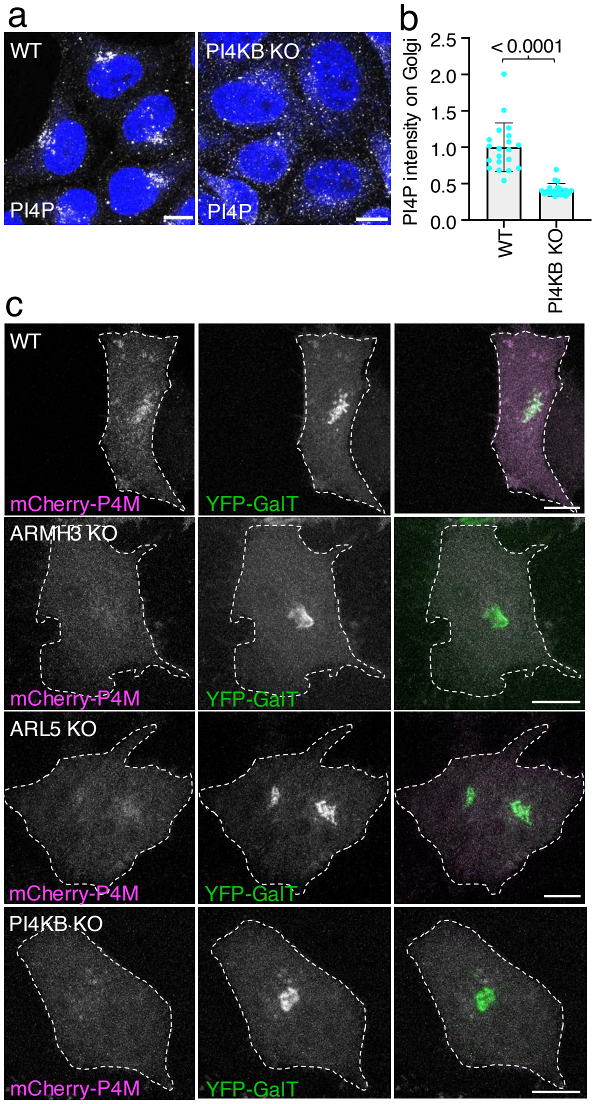

Fig. S6: Golgi PI4P is dependent on PI4KB, ARMH3, and ARL5.

(a) Immunofluorescence microscopy of WT and PI4KB-KO HeLa cells stained for endogenous PI4P. Single-channel images are shown in grayscale with nuclei (DAPI) in blue. (b) Quantification of PI4P intensity at the Golgi complex relative to the cytosol from experiments such as that shown in panel a. The graph represents the mean ± SD of values from 20 cells, as shown on the graph. The statistical significance of the differences was calculated by unpaired t-test (two-tailed). *P*-value is indicated on the graph. (c) Live-cell imaging of WT, ARMH3-KO, ARL5-KO, and PI4KB-KO HeLa cells expressing the PI4P biosensor mCherry-P4M (magenta) and Golgi marker YFP-GalT (green). Scale bars: 10 μm. Source data are provided in the Source Data file.

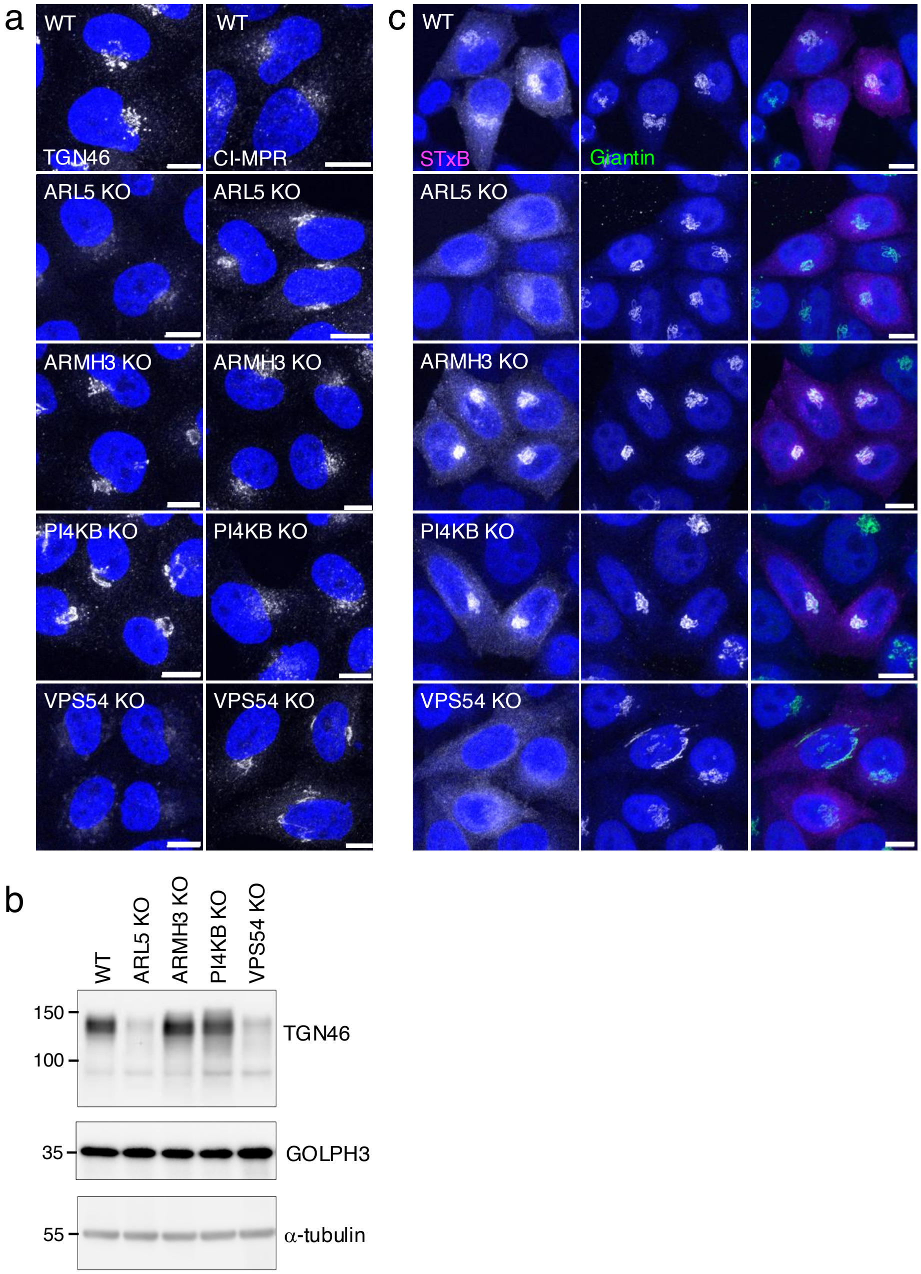

Fig. S7: The ARL5-ARMH3-PI4KB axis is not involved in retrograde transport.

(a) Immunofluorescence microscopy of WT and the indicated KO HeLa cells immunostained for endogenous TGN46 or CI-MPR. Images are shown in grayscale with nuclei (DAPI) in blue. Scale bars: 10 µm. Notice the disappearance of TGN46 from the Golgi complex and the higher intensity of CI-MPR in ARL5-KO and VPS54-KO cells, and the lack of an effect of ARMH3-KO and PI4K Β -KO on these proteins. (b) Immunoblot analysis of endogenous TGN46, GOLPH3, and α -tubulin (loading control) in WT and the indicated KO HeLa cells. The positions of molecular mass markers (kDa) are indicated on the left. Notice the decreased levels of TGN46 in

ARL5-KO and VPS54-KO cells, but not in ARMH3-KO and PI4KΒ-KO cells. (c) Live WT and the indicated KO Hela cells were incubated for 15 min with Cy3-STxB (magenta) and chased for 1 h in regular culture medium at 37 °C. Cells were then fixed and stained for endogenous giantin (green) and DAPI (blue). Scale bars: 10 μm. Notice the decreased delivery of internalized STxB to the Golgi complex in ARL5-KO and VPS54-KO cells, but not in ARMH3-KO and PI4KΒ-KO cells.

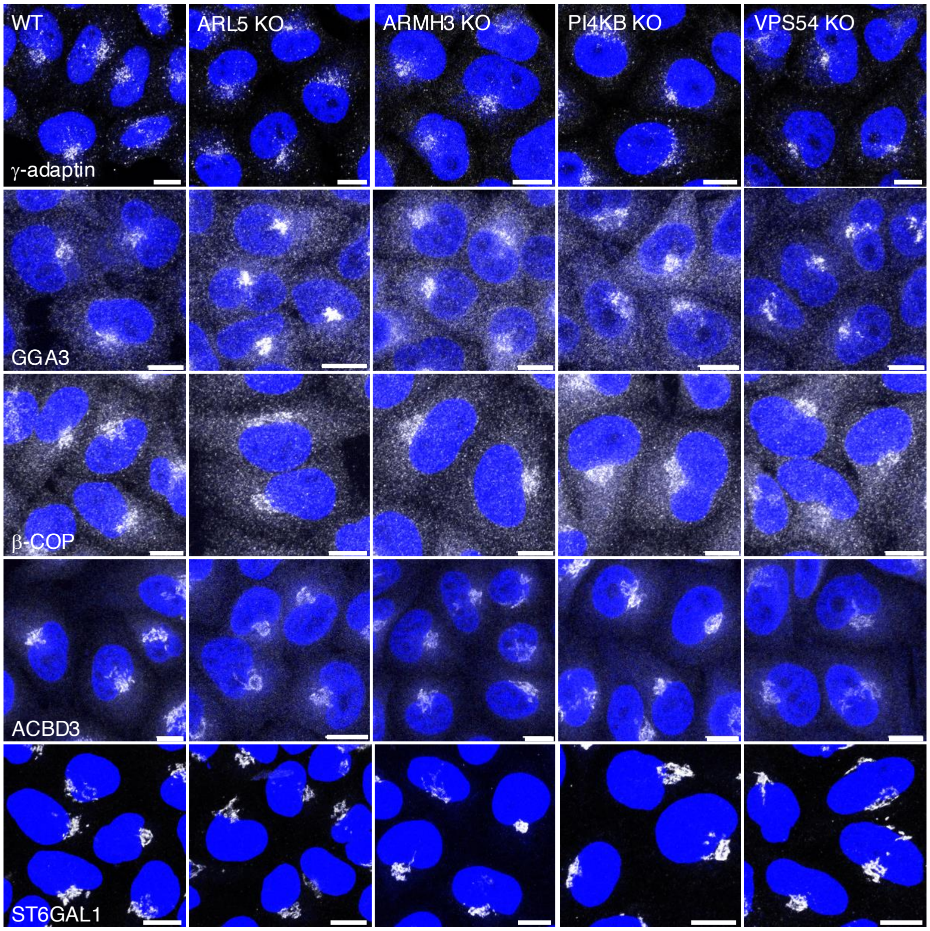

Fig. S8: Immunostaining of Golgi proteins in Hela cells with KO of ARL5, ARMH3, PI4KB or VPS54.

WT and the indicated KO HeLa cells were stained for γ1-adaptin (subunit of the AP-1 complex), GGA3, β-COP (subunit of the COPI complex), ACBD3, ST6GAL1, and nuclei (DAPI), and analyzed by confocal fluorescence microscopy. Images are shown in grayscale with nuclei in blue. Scale bars: 10 μm. Notice the unchanged staining pattern of all these proteins in the KO cell lines.

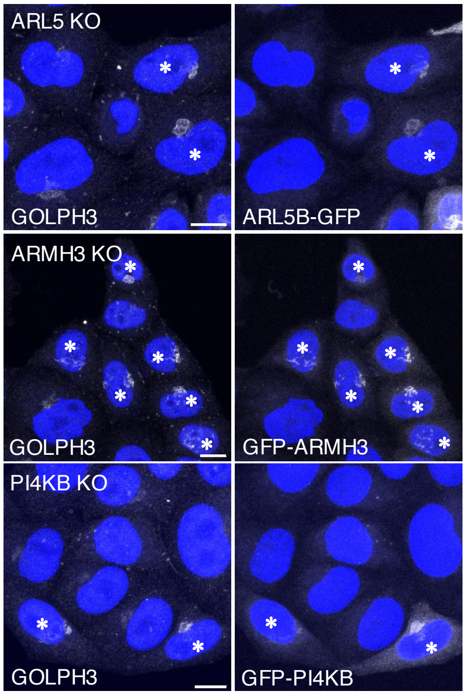

Fig. S9: GOLPH3 localization at the Golgi complex is dependent on ARL5, ARMH3, and PI4KB.

Immunofluorescence microscopy of ARL5-, ARMH3-, and PI4KB-KO HeLa cells transiently expressing ARL5B-GFP, GFP-ARMH3, and GFP-PI4KB, respectively, fixed and stained for endogenous GOLPH3. Cells expressing the rescue constructs are indicated with an asterisk. Scale bars: 10 μm. Note that GOLPH3 localization to the Golgi is restored in cells expressing the rescue constructs.
