# Supplementary material for: ARMH3 is an ARL5 effector that promotes PI4KB-catalyzed PI4P synthesis at the trans-Golgi network
Source: Nat Commun. 2024 Nov 23;15:10168. doi: 10.1038/s41467-024-54410-y (PMC11585589; doi:10.1038/s41467-024-54410-y)
Supplement: Supplementary file 2 — Description of Additional Supplementary Files [file 41467_2024_54410_MOESM2_ESM.pdf]

Description of Additional Supplementary Files

File name: Supplementary Data 1

Description: Proteins identified by mass spectrometry in ARL5 MitoID.

ARL5A_QL/Ctrl: MTS-BioID2-ARL5A-Q70L/MTS-BioID2, ARL5B_TN/Ctrl: MTS-BioID2-ARL5B-T30N/MTS-BioID2, ARL5A_TN/Ctrl: MTS-BioID2-ARL5A-T30N/MTS-BioID2, ARL5B_QL/Ctrl: MTS-BioID2-ARL5B-Q70L/MTS-BioID2

File name: Supplementary Data 2

Description: Proteins identified by mass spectrometry in ARMH3 (also known as c10orf76) MitoID.

MitoID_c10orf76/MitoID_Ctrl: MTS-BioID2-ARMH3/MTS-BioID2
