# Supplementary material for: ARMH3 is an ARL5 effector that promotes PI4KB-catalyzed PI4P synthesis at the trans-Golgi network
Source: Nat Commun. 2024 Nov 23;15:10168. doi: 10.1038/s41467-024-54410-y (PMC11585589; doi:10.1038/s41467-024-54410-y)
Supplement: Supplementary file 5 — Reporting Summary [file 41467_2024_54410_MOESM5_ESM.pdf]

nature portfolio

Corresponding author(s): Juan S. Bonifacino

Last updated by author(s): Oct 21, 2024

Reporting Summary

Nature Portfolio wishes to improve the reproducibility of the work that we publish. This form provides structure for consistency and transparency in reporting. For further information on Nature Portfolio policies, see our Editorial Policies and the Editorial Policy Checklist.

Statistics

For all statistical analyses, confirm that the following items are present in the figure legend, table legend, main text, or Methods section.

n/a	Confirmed	
☐	☒	The exact sample size (*n*) for each experimental group/condition, given as a discrete number and unit of measurement
☐	☒	A statement on whether measurements were taken from distinct samples or whether the same sample was measured repeatedly
☐	☒	The statistical test(s) used AND whether they are one- or two-sided<br>*Only common tests should be described solely by name; describe more complex techniques in the Methods section.*
☒	☐	A description of all covariates tested
☐	☒	A description of any assumptions or corrections, such as tests of normality and adjustment for multiple comparisons
☐	☒	A full description of the statistical parameters including central tendency (e.g. means) or other basic estimates (e.g. regression coefficient) AND variation (e.g. standard deviation) or associated estimates of uncertainty (e.g. confidence intervals)
☐	☒	For null hypothesis testing, the test statistic (e.g. *F*, *t*, *r*) with confidence intervals, effect sizes, degrees of freedom and *P* value noted<br>*Give P values as exact values whenever suitable.*
☒	☐	For Bayesian analysis, information on the choice of priors and Markov chain Monte Carlo settings
☒	☐	For hierarchical and complex designs, identification of the appropriate level for tests and full reporting of outcomes
☐	☒	Estimates of effect sizes (e.g. Cohen's *d*, Pearson's *r*), indicating how they were calculated

Our web collection on statistics for biologists contains articles on many of the points above.

Software and code

Policy information about availability of computer code

Data collection	Cells were imaged by confocal microscopy (LSM780 or LSM880, Carl Zeiss) with an oil-immersion 63×/1.40 NA Plan-Apochromat Oil DIC M27 objective lens (Carl Zeiss), or by structured illumination microscopy (SIM) (Zeiss Elyra PS.1, Carl Zeiss) with a Plan-Apochromat 63×/1.4 NA objective lens at room temperature. For SIM, three orientation angles of the excitation grid with five phases each were acquired for each z plane. Images were then reconstructed with the SIM module in Zeiss ZEN Black software (version 14.0.27.201) using the automatic setting. Z-stack calibration images of a multi-speck bead slide (Carl Zeiss AG, 1783–455) were acquired with equivalent microscope settings, reconstructed as above, and used to correct chromatic aberration by applying affine fit of experimental images to bead calibration images using the channel alignment processing module. Maximum intensity projections were generated with Zeiss ZEN Black software, and final composite images were created using ImageJ/Fiji (https://fiji.sc/). Blots images were acquired on a ChemiDoc (Bio-Rad) with Image Lab software (Bio-Rad). Yeast two and three hybrid images were obtained by scanning with EPSON scanner.
Data analysis	Fiji/ImageJ: https://fiji.sc/ RRID:SCR_002285 v2.9.0<br>Image Lab, Bio-Rad: https://www.bio-rad.com/ja-jp/product/image-lab-software?ID=KRE6P5E8Z RRID:SCR_014210<br>GraphPad Prism: http://www.graphpad.com/ RRID:SCR_002798 v9.5.0<br>SnapGene, Dotmatics: http://www.snapgene.com/ RRID:SCR_015052 v6.2.1<br>BioRender: https://www.biorender.com/ RRID:SCR_018361 v2023<br>Zen black, Zeiss: https://www.micro-shop.zeiss.com/en/us/softwarefinder/software-categories/zenblack/ v14.0

For manuscripts utilizing custom algorithms or software that are central to the research but not yet described in published literature, software must be made available to editors and reviewers. We strongly encourage code deposition in a community repository (e.g. GitHub). See the Nature Portfolio guidelines for submitting code & software for further information.

Data

Policy information about availability of data

All manuscripts must include a data availability statement. This statement should provide the following information, where applicable:

- Accession codes, unique identifiers, or web links for publicly available datasets
- A description of any restrictions on data availability
- For clinical datasets or third party data, please ensure that the statement adheres to our policy

Reagents generated in this study are available upon request. All data are available in the main text or the supplementary materials. Further information and requests for resources and reagents should be directed to the lead contact, Juan S. Bonifacino (juan.bonifacino@nih.gov). Accession numbers are listed in the key resources table. Microscopy data reported in this paper will be shared by the lead contact upon request.

Research involving human participants, their data, or biological material

Policy information about studies with human participants or human data. See also policy information about sex, gender (identity/presentation), and sexual orientation and race, ethnicity and racism.

Reporting on sex and gender	No human subjects were used in this study.
Reporting on race, ethnicity, or other socially relevant groupings	No human subjects were used in this study.
Population characteristics	N/A
Recruitment	N/A
Ethics oversight	N/A

Note that full information on the approval of the study protocol must also be provided in the manuscript.

Field-specific reporting

Please select the one below that is the best fit for your research. If you are not sure, read the appropriate sections before making your selection.

[x] Life sciences   [ ] Behavioural & social sciences   [ ] Ecological, evolutionary & environmental sciences

For a reference copy of the document with all sections, see nature.com/documents/nr-reporting-summary-flat.pdf

Life sciences study design

All studies must disclose on these points even when the disclosure is negative.

Sample size	The total number of samples (n) analyzed in each experiment is indicated in the figure legends. No statistical methods were used to predetermine sample sizes, but our sample sizes are like those reported in previous publications.
Data exclusions	Data exclusion was not performed in this study.
Replication	Multiple independent experiments were carried out using several sample replicates as detailed in the figure legends.
Randomization	Data collection was not randomized. Data distribution was assumed to be normal, but this was not formally tested.
Blinding	Data collection and analysis were not performed blind to the conditions of the experiments.

Reporting for specific materials, systems and methods

We require information from authors about some types of materials, experimental systems and methods used in many studies. Here, indicate whether each material, system or method listed is relevant to your study. If you are not sure if a list item applies to your research, read the appropriate section before selecting a response.

April 2023

Materials & experimental systems

n/a	Involved in the study
☐	☒ Antibodies
☐	☒ Eukaryotic cell lines
☒	☐ Palaeontology and archaeology
☒	☐ Animals and other organisms
☒	☐ Clinical data
☒	☐ Dual use research of concern
☒	☐ Plants

Methods

n/a	Involved in the study
☒	☐ ChIP-seq
☒	☐ Flow cytometry
☒	☐ MRI-based neuroimaging

Antibodies

Antibodies used

chicken anti-BioID2, used 1:1,000 for IF (BioFront Technologies, BID2-CP-100)
mouse HRP-conjugated anti-GFP, used 1:2,000 for WB (Miltenyi Biotec, 130-091-833)
rat anti-HA epitope (Roche, 12158167001) used 1:1,000 for IF, 1:5,000 for WB,
rabbit anti-GFP used 1:1,000 for IF (Invitrogen, A-11122)
mouse anti-PI4KB, used 1:200 for IF, 1:1,000 for WB (BD Biosciences,611817)
mouse HRP-conjugated anti-α-tubulin, used 1:5,000 for WB (Santa Cruz Biotechnology, sc-32293 HRP)
mouse anti-PI4P used 1:200 for IF (Echelon Biosciences, Z-P004)
rabbit anti-giantin, used 1:500 for IF (Abcam, ab80864)
rabbit anti-ARMH3, used 1:200 for WB (Invitrogen, PA5-62264)
rabbit anti-GOLPH3, used 1:200 for IF, 1:1,000 for WB (Abcam, ab98023)
mouse anti-CI-MPR, used 1:200 for IF (Abcam, ab2733)
sheep anti-TGN46, used 1:200 for IF, 1:5,000 for WB (Bio-Rad, AHP500G)
mouse anti-γ1-adaptin, used 1:200 for IF (BD Biosciences, 610385)
rabbit anti-β-COP, used 1:200 for IF (Invitrogen, PA1-061)
mouse anti-GGA3, used 1:200 for IF (BD Biosciences, 612310)
mouse anti-ACBD3, used 1:200 for IF (Sigma-Aldrich, WH0064746M1)
goat anti-ST6GAL1, used 1:200 for IF (R&D Systems, AF5924)
mouse anti-LAMP1 to detect fully glycosylated protein, used 1:5,000 for WB (DSHB, H4A3-C)
rabbit anti-LAMP1 to detect both glycosylated and deglycosylated protein, used 1:1000 for WB (Cell Signaling Technology, 9091)
Alexa Fluor 555 goat anti-chicken IgY, used 1:1,000 for IF (Invitrogen, A-21437)
Alexa Fluor 488 donkey anti-rabbit IgG, used 1:1,000 for IF (Invitrogen, A-21206)
Alexa Fluor 555 donkey anti-mouse IgG, used 1:1000 for IF (Invitrogen, A-31570)
Alexa Fluor 647 donkey anti-rat IgG , used 1:1,000 for IF (Invitrogen, A-48272)
Alexa Fluor 488 donkey anti-mouse IgG, used 1:1,000 for IF (Invitrogen, A-21202)
Alexa Fluor 546 donkey anti-rabbit IgG, used 1:1,000 for IF (Invitrogen, A-10040)
HRP-conjugated donkey anti-sheep, used 1:2,000 for WB (R&D Systems, HAF016)
HRP-conjugated goat anti-rabbit IgG, used 1:2,000 for WB (Jackson Immuno Research, 111-035-003)
HRP-conjugated goat anti-rat IgG, used 1:2,000 for WB (Jackson Immuno Research, 112-035-143)
HRP-conjugated goat anti-mouse IgG, used 1:2000 for WB (Jackson Immuno Research, 715-035-150)

Validation

All antibodies are from commercially available sources and have been validated by the manufacturer with supporting publications found on manufacturer websites.

Eukaryotic cell lines

Policy information about cell lines and Sex and Gender in Research

Cell line source(s)	HEK293T (ATCC, CRL-3216) and HeLa (ATCC, CCL-2) cells were used in this study.
Authentication	Specific KO mutations were confirmed via western blotting using the corresponding antibodies.
Mycoplasma contamination	The cell lines were not tested for mycoplasma contamination. But the cells were cultured in media supplemented with Mycozap-CL plus (VZA-2012) for prevention of contamination of mycoplasma, and DAPI-staining did not show any sign of mycoplasma contamination throughout this study.
Commonly misidentified lines (See ICLAC register)	No commonly misidentified lines were used in this study.

Plants

Seed stocks	No plants were used in this study.
Novel plant genotypes	N/A
Authentication	N/A
