# Supplementary material for: ARMH3 is an ARL5 effector that promotes PI4KB-catalyzed PI4P synthesis at the trans-Golgi network
Source: Nat Commun. 2024 Nov 23;15:10168. doi: 10.1038/s41467-024-54410-y (PMC11585589; doi:10.1038/s41467-024-54410-y)
Supplement: Supplementary file 7 — Source Data [file 41467_2024_54410_MOESM7_ESM.zip › Source Data_blots.pptx]

## Slide 1
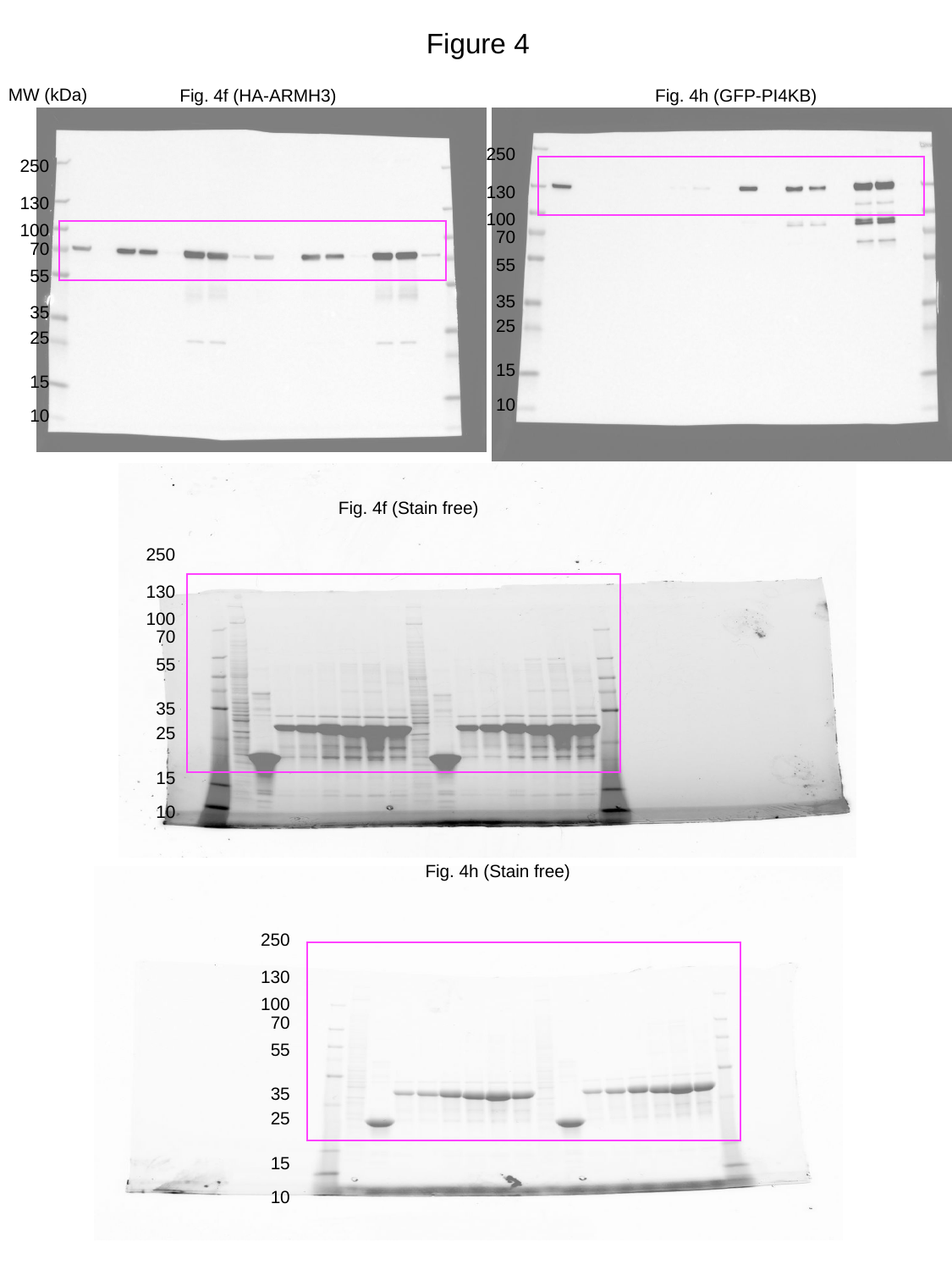

Figure 4
MW (kDa)
Fig. 4f (HA-ARMH3)
Fig. 4h (GFP-PI4KB)
250
250
130
130
100
100
70
70
55
55
35
35
25
25
15
15
10
10
Fig. 4f (Stain free)
250
130
100
70
55
35
25
15
10
Fig. 4h (Stain free)
250
130
100
70
55
35
25
15
10

## Slide 2
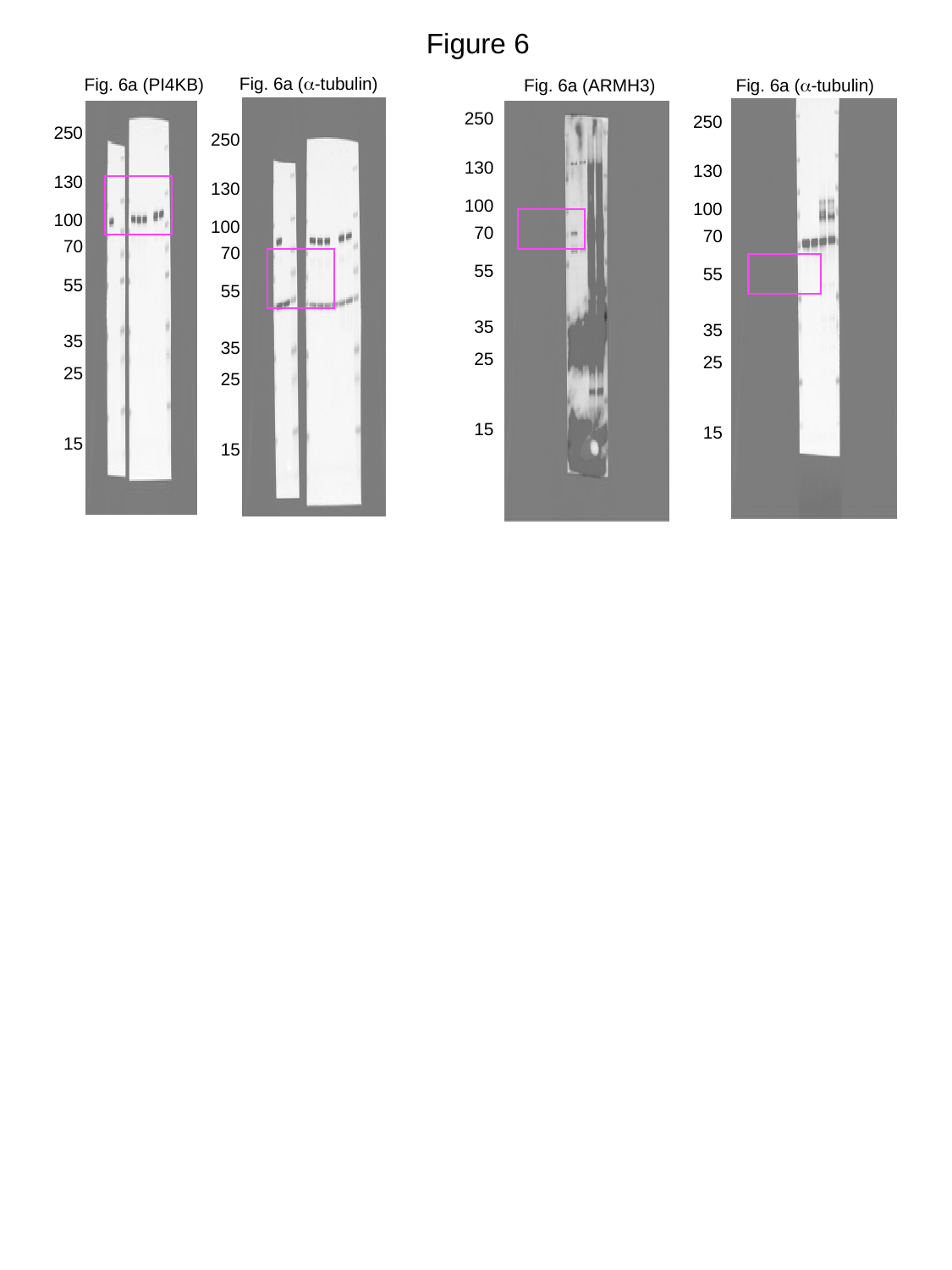

Figure 6
Fig. 6a (a-tubulin)
Fig. 6a (PI4KB)
Fig. 6a (ARMH3)
Fig. 6a (a-tubulin)
250
250
250
250
130
130
130
130
100
100
100
100
70
70
70
70
55
55
55
55
35
35
35
35
25
25
25
25
15
15
15
15

## Slide 3
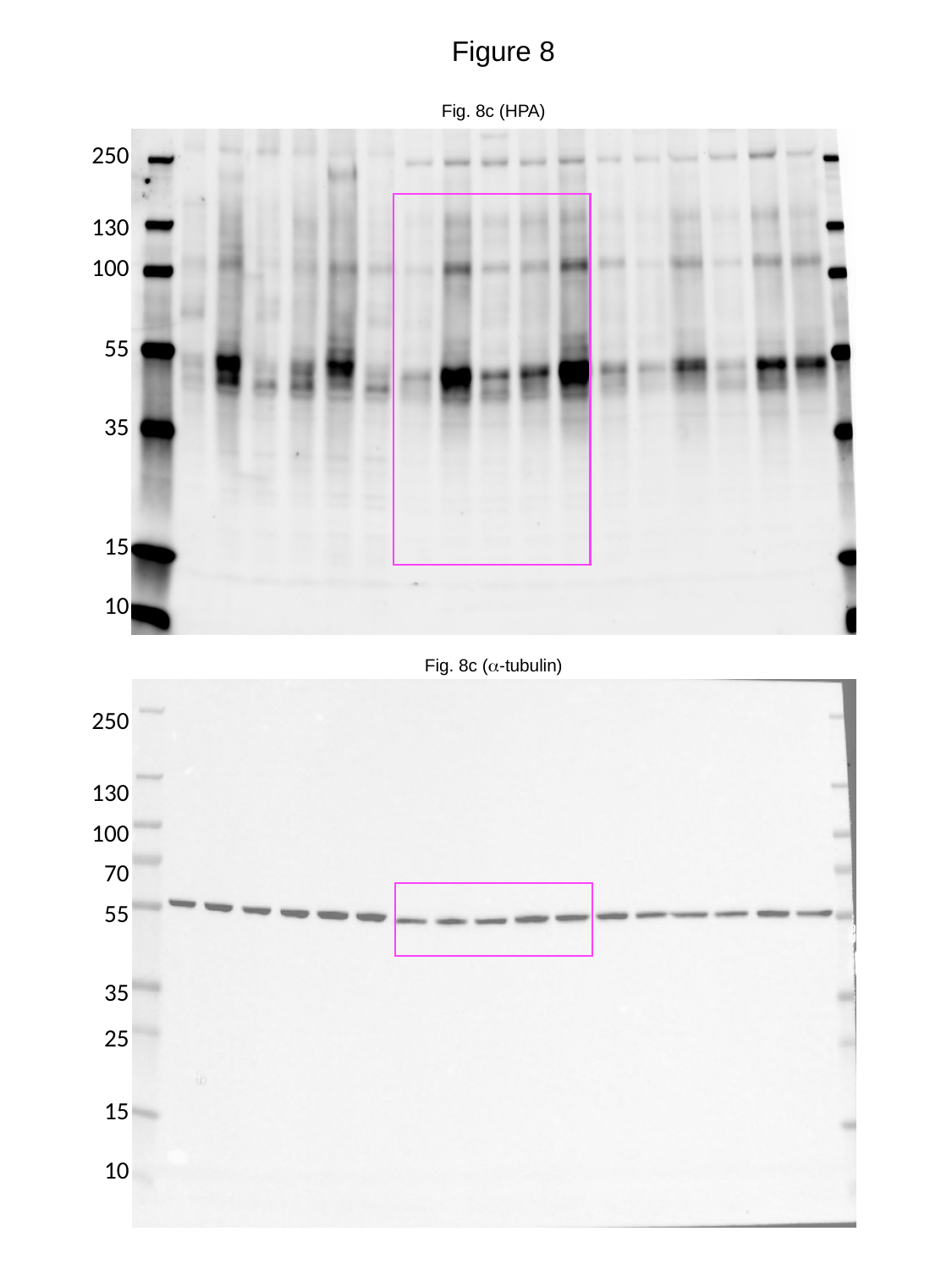

Figure 8
Fig. 8c (HPA)
250
130
100
55
35
15
10
Fig. 8c (a-tubulin)
250
130
100
70
55
35
25
15
10

## Slide 4
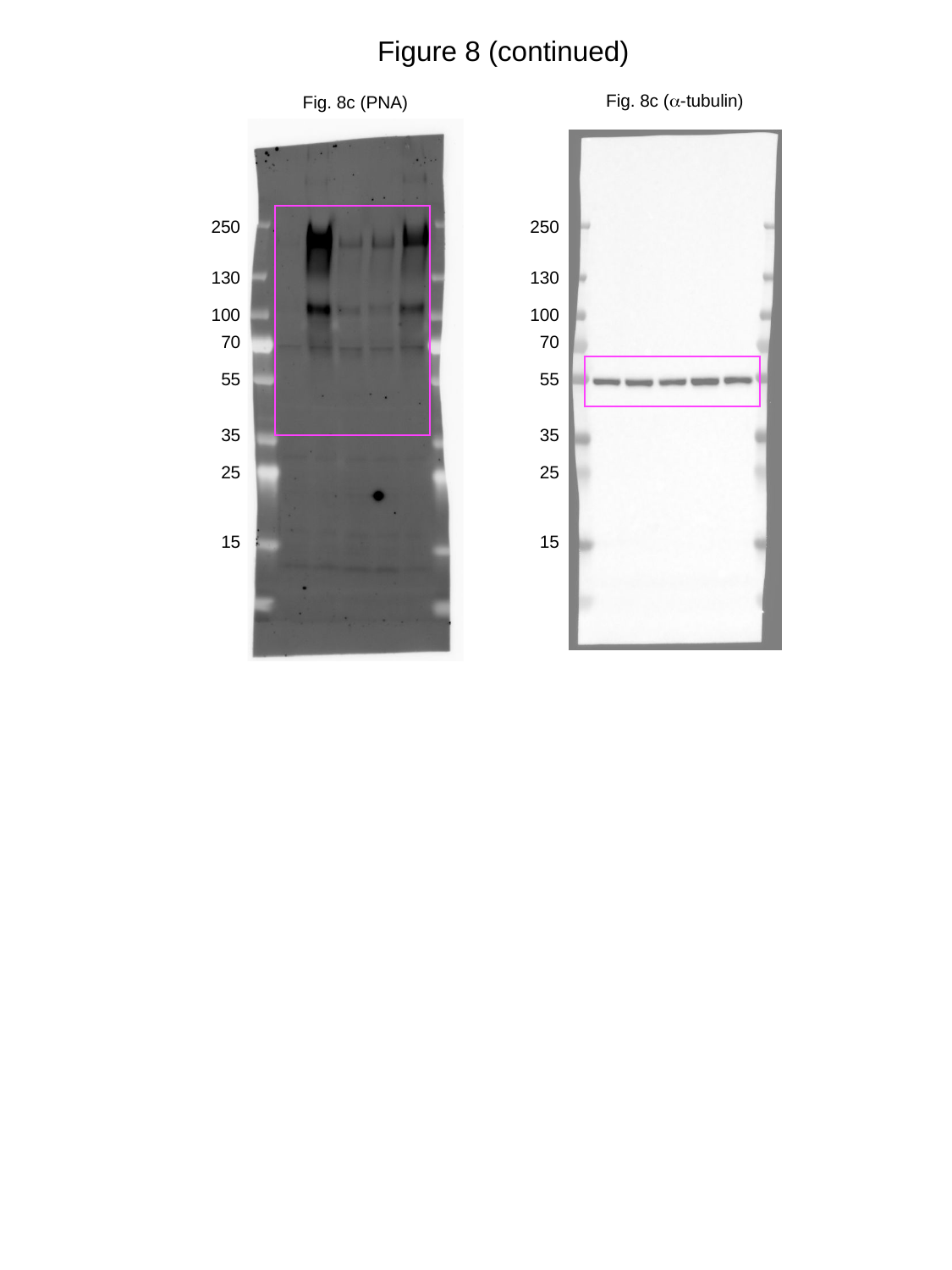

Figure 8 (continued)
Fig. 8c (a-tubulin)
Fig. 8c (PNA)
250
250
130
130
100
100
70
70
55
55
35
35
25
25
15
15

## Slide 5
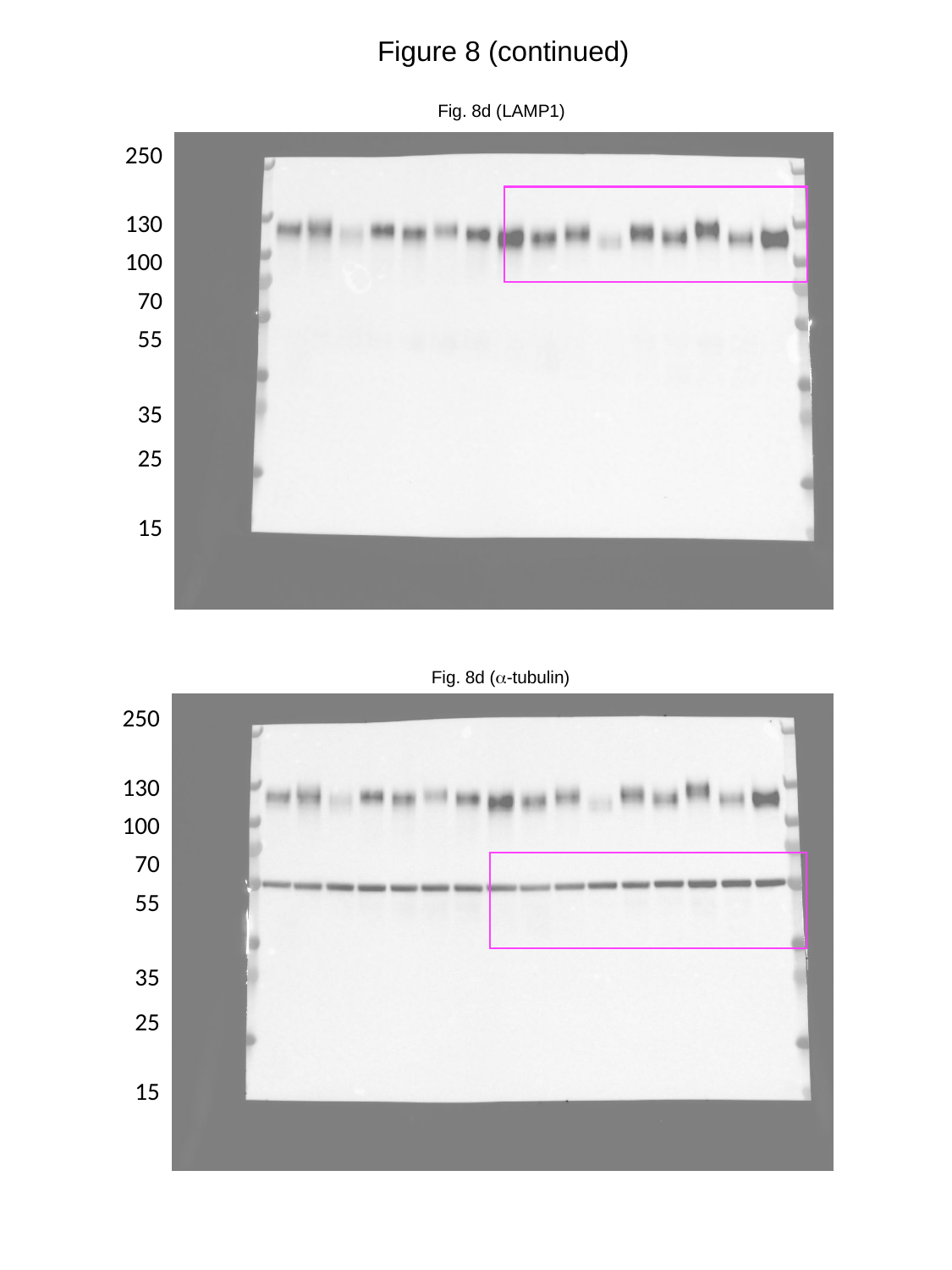

Figure 8 (continued)
Fig. 8d (LAMP1)
250
130
100
70
55
35
25
15
Fig. 8d (a-tubulin)
250
130
100
70
55
35
25
15

## Slide 6
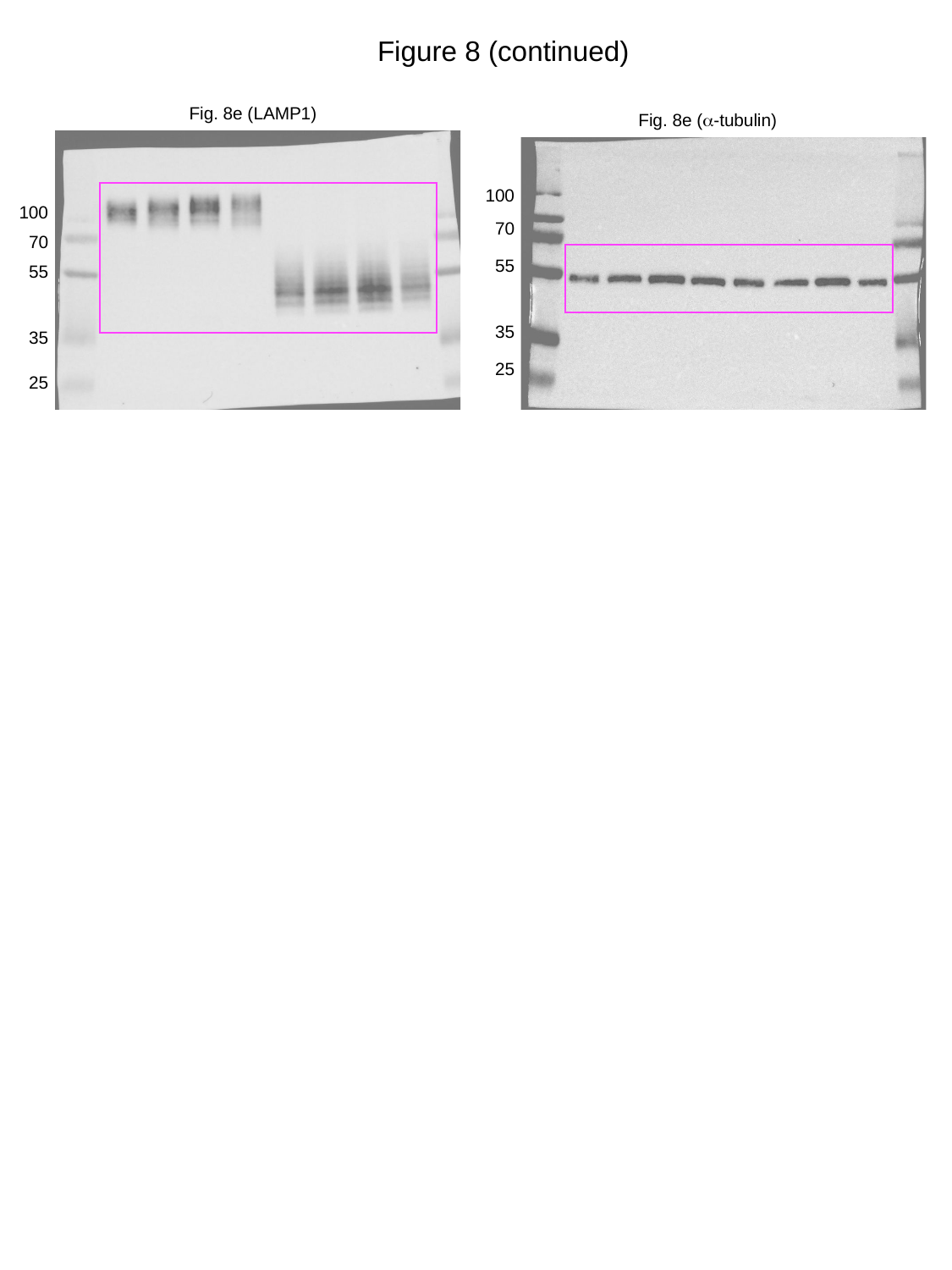

Figure 8 (continued)
Fig. 8e (LAMP1)
Fig. 8e (a-tubulin)
100
100
70
70
55
55
35
35
25
25

## Slide 7
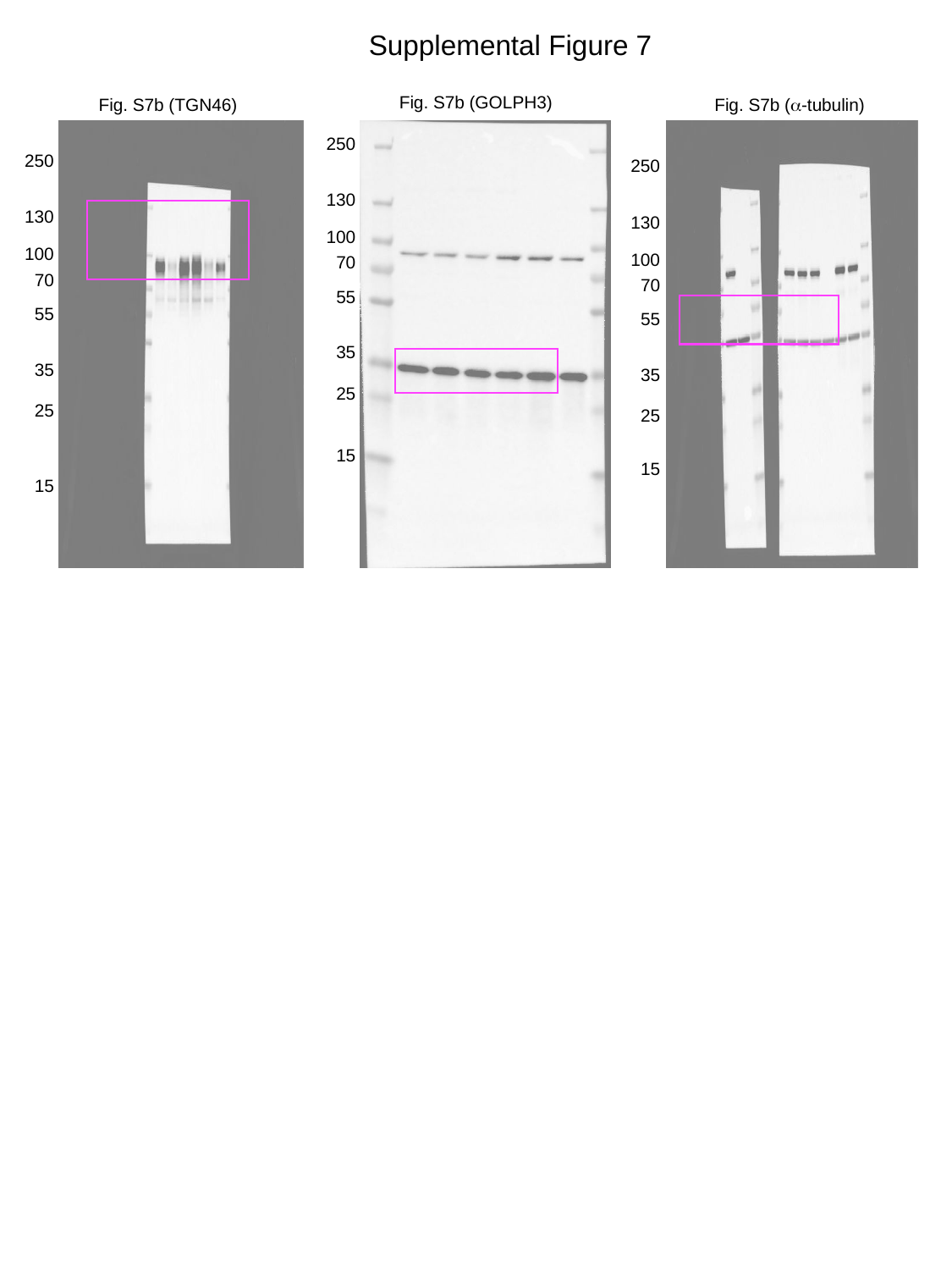

Supplemental Figure 7
Fig. S7b (GOLPH3)
Fig. S7b (TGN46)
Fig. S7b (a-tubulin)
250
250
250
130
130
130
100
100
100
70
70
70
55
55
55
35
35
35
25
25
25
15
15
15
